# Insights into high‐risk or unusual ARIA: manifestations and individualized approaches to management through cases from DIAN‐TU gantenerumab open‐label study

**DOI:** 10.1002/alz.094833

**Published:** 2025-01-09

**Authors:** Alireza Atri, Jorge J. Llibre‐Guerra, David B. Clifford, Eric McDade, Yan Li, Susan Mills, Anna Santacruz, Guoqiao Wang, Charlene B Supnet, Tammie L.S. Benzinger, Brian A. Gordon, Laura Ibanez, Jakub Wojtowicz, Gregory Klein, Monika Baudler, Geoffrey A. Kerchner, Randall J. Bateman

**Affiliations:** ^1^ Banner Health, Phoenix, AZ USA; ^2^ Knight Alzheimer Disease Research Center, St Louis, MO USA; ^3^ Washington University in St. Louis, School of Medicine, St. Louis, MO USA; ^4^ Knight Alzheimer Disease Research Center, St. Louis, MO USA; ^5^ Washington University in St. Louis School of Medicine, St. Louis, MO USA; ^6^ Washington University in St. Louis, St. Louis, MO USA; ^7^ F. Hoffmann‐La Roche Ltd., Pharma Development Safety Risk Management, Basel Switzerland; ^8^ Roche Pharma Research and Early Development, Neuroscience and Rare Diseases Biomarkers, Basel Switzerland; ^9^ F. Hoffmann‐La Roche Ltd, Basel Switzerland

## Abstract

**Background:**

Amyloid‐related Imaging Abnormalities (ARIA) are side effects of beta‐amyloid plaque‐lowering monoclonal antibody drugs (APLmAbs). Understanding of ARIA mechanisms, risks, nature, evolution and optimal approaches to mitigation and management remains incomplete. Sporadic Alzheimer’s Disease (AD) trials support ARIA risk factors including higher APLmAb doses/exposures, ApoE4‐carrier status, and presence/severity of microhemorrhages (MCH)/superficial siderosis. ARIA can present and evolve across a wide spectrum of radiographic and clinical profiles and be mitigated through patient selection, clinical/MRI surveillance/detection, monitoring and management practices. Through atypical cases selected from participants with dominantly‐inherited AD (Cognitively unimpaired/CU or MCI stages) treated with an APLmAb, insights are shared into unusual manifestations or high‐risk ARIA (e.g. multiple recurrences, multifocal or radiologically severe) and approaches to participant‐centered shared decision‐making and individualized management.

**Method:**

Clinical and imaging course, management and insights from 5 selected cases with unusual profiles or high‐risk ARIA‐E (± ARIA‐H) from the DIAN‐TU Gantenerumab Open‐label trial will be presented.

**Result:**

Case#1: “Recurrent ARIA‐E over 6 years” with 6 asymptomatic unifocal/multifocal ARIA‐E events over six‐years in CU participant (CDR 0.0, MMSE 29, Expected‐Year‐of‐Onset AD symptoms [EYO]+2.5 years, e4‐heterozygous). Case#2: “Recurrent ARIA‐E with accumulating +32 MCHs” in participant with MCI (CDR 0.5, MMSE 28, EYO+6 years, e4‐heterozygous) treated with oral steroids. Case#3 and Case#4: “Late‐Onset” ARIA‐E with development of ARIA‐E late in the course of treatment with high‐dose gantenerumab (e.g. years into treatment initiation/titration and tolerance of 1500mg Q2W for >6 months) in two CU participants (CDR 0.0, MMSE 30, ∼EYO‐6 years) with no previous history of ARIA‐E. Case#3 (e4‐heterozygous) developed unifocal (13mm) asymptomatic ARIA‐E, continued on treatment with resolution of ARIA‐E. Case#4 (e4‐non‐carrier) developed radiographically severe multifocal and multilobar, mildly symptomatic ARIA‐E (+1 ARIA‐H superficial siderosis); gantenrumab was held but ARIA increased, and then resolved after IV pulse‐steroids with oral taper. Case#5 “Recurrent ARIA‐E in same area in CU participant with EYO+10 years (CDR 0.0, MMSE 29, e4‐heterozygous).

**Conclusion:**

These illustrative cases may provide insights into unusual profiles (e.g. Late‐onset/“delayed” or CU with +EYO) or high‐risk ARIA‐E and suggest potential approaches and considerations for clinicians and trialists to individualize APLmAb treatment and ARIA detection, monitoring and management.

